# Life-cycle of *Oxyspirura petrowi* (Spirurida: Thelaziidae), an eyeworm of the northern bobwhite quail (*Colinus virginianus*)

**DOI:** 10.1186/s13071-019-3802-3

**Published:** 2019-11-21

**Authors:** Aravindan Kalyanasundaram, Matthew Z. Brym, Kendall R. Blanchard, Cassandra Henry, Kalin Skinner, Brett J. Henry, Jessica Herzog, Alyssa Hay, Ronald J. Kendall

**Affiliations:** The Wildlife Toxicology Laboratory, Texas Tech University, Lubbock, TX 79409-3290 USA

**Keywords:** Eyeworm, Bobwhite, Crickets, Life-cycle, Intermediate, Definitive

## Abstract

**Background:**

*Oxyspirura petrowi* (Spirurida: Thelaziidae), a heteroxenous nematode of birds across the USA, may play a role in the decline of the northern bobwhite (*Colinus virginianus*) in the Rolling Plains Ecoregion of West Texas. Previous molecular studies suggest that crickets, grasshoppers and cockroaches serve as potential intermediate hosts of *O. petrowi*, although a complete study on the life-cycle of this nematode has not been conducted thus far. Consequently, this study aims to improve our understanding of the *O. petrowi* life-cycle by experimentally infecting house crickets (*Acheta domesticus*) with *O. petrowi* eggs, feeding infected crickets to bobwhite and assessing the life-cycle of this nematode in both the definitive and intermediate hosts.

**Methods:**

*Oxyspirura petrowi* eggs were collected from gravid worms recovered from wild bobwhite and fed to house crickets. The development of *O. petrowi* within crickets was monitored by dissection of crickets at specified intervals. When infective larvae were found inside crickets, parasite-free pen-raised bobwhite were fed four infected crickets each. The maturation of *O. petrowi* in bobwhite was monitored through fecal floats and bobwhite necropsies at specified intervals.

**Results:**

In this study, we were able to infect both crickets (*n* = 45) and bobwhite (*n* = 25) with *O. petrowi* at a rate of 96%. We successfully replicated and monitored the complete *O. petrowi* life-cycle *in vivo*, recovering embryonated *O. petrowi* eggs from the feces of bobwhite 51 days after consumption of infected crickets. All life-cycle stages of *O. petrowi* were confirmed in both the house cricket and the bobwhite using morphological and molecular techniques.

**Conclusions:**

This study provides a better understanding of the infection mechanism and life-cycle of *O. petrowi* by tracking the developmental progress within both the intermediate and definitive host. To our knowledge, this study is the first to fully monitor the complete life-cycle of *O. petrowi* and may allow for better estimates into the potential for future epizootics of *O. petrowi* in bobwhite. Finally, this study provides a model for experimental infection that may be used in research examining the effects of *O. petrowi* infection in bobwhite.

## Background

The eyeworm (*Oxyspirura petrowi*) is a heteroxenous nematode found in birds of the USA. In the USA, it was first reported in Galliformes and Passeriformes in Michigan during 1937 [1]. Since then, it has been identified in numerous other species from these orders, including the lesser-prairie chicken (*Tympanuchus pallidicinctus*) [2], northern cardinal (*Cardinalis cardinalis*) [3], northern mockingbird (*Mimus polyglottos*), curve-billed thrasher (*Toxostoma curvirostre*) [4], Gambel’s quail (*Callipepla* gambelii) [5], scaled quail (*Callipepla squamata*) and northern bobwhite quail (*Colinus virginianus*; hereafter, bobwhite) [6]. *Oxyspirura petrowi* has gained particular notoriety in the Rolling Plains Ecoregion of West Texas, as this area is reported to be the epicenter of infection [7]. The West Texas Rolling Plains is also considered to be a stronghold of bobwhite hunting [5], which provides an important source of seasonal revenue for many local communities [8]. Unfortunately, bobwhite populations throughout the West Texas Rolling Plains have been declining, with *O. petrowi* infection being purported as a potential mechanism contributing to this decline [9–11].

In the Rolling Plains, there have been extensive studies investigating the effects of *O. petrowi* on bobwhite and surveys have identified it to be highly prevalent throughout the ecoregion [7, 12]. For instance, Dunham et al. [13] found 58.7% of adult bobwhite across 29 counties in the Rolling Plains to be infected with *O. petrowi*, while others have found some areas with a prevalence of 100% [10, 11]. *Oxyspirura petrowi* is typically found on the surface of the eye, under the nictitating membrane, as well as in the lacrimal ducts and other glands of the eye [9]. *Oxyspirura petrowi* infections have been correlated with inflammation of the lacrimal ducts, keratitis and lesions on the Harderian gland [9, 14], leading to suspicions that infection may adversely affect bobwhite [9–11]. Pathological investigations have further increased concerns of *O. petrowi* infection, as the Harderian gland is associated with immune function [15]; although more research is needed to elucidate links between infection and these immune system processes. Moreover, phylogenetic analyses have revealed the close relation of *O. petrowi* to the human eyeworm (*Loa loa*) [16, 17] and the human and carnivore eyeworm (*Thelazia callipaeda*) [17], which have been associated with impaired vision [18] and ulcerative keratitis [19] in their hosts, respectively. While the phylogenetic similarity of *O. petrowi* to *L. loa* and *T. callipaeda* does not necessarily imply similar pathology, the documented pathological consequences of *O. petrowi* infection alongside reports of bobwhite flying into stationary objects [20, 21] suggest that impaired vision may occur in birds infected with this parasite.

Although many studies have investigated the effects of *O. petrowi* on bobwhite, there is a lack of research into how bobwhite are infected with this nematode. Thus, understanding the full life-cycle of *O. petrowi* could provide valuable insight to transmission dynamics in wild bobwhite. *Thelazia callipaeda*, for example, utilizes a fly as an intermediate host to transmit third-stage larvae to the definitive host [19, 22, 23], while the chicken eyeworm (*Oxyspirura mansoni*) utilizes the Surinam cockroach (*Pycnoscelus surinamensis*) for this purpose [24]. It is known that *O. petrowi* also requires an intermediate host and multiple insect species have been identified in this capacity, including the differential grasshopper (*Melanoplus differentialis*), wood cockroach (*Parcoblatta* spp.) and red-legged grasshopper (*Melanoplus femurrubrum*) [25]. Furthermore, pioneering research by Kistler et al. [26] reported the plains lubber grasshopper (*Brachystola magna*) as an intermediate host, with *O. petrowi* larvae being found in the body cavity of various specimens. Kistler et al. [26] confirmed the identity of *O. petrowi* in *B. magna* by experimentally infecting house crickets (*Acheta domesticus*; hereafter, cricket) with *O. petrowi* and comparing the morphology of third-stage larvae recovered from both orthopterans. Kistler et al. [26] also fed infected *B. magna* to pen-raised bobwhite, recovering sexually mature *O. petrowi* from these birds. However, Kistler et al. [26] achieved only low (16.6%) infection rates in crickets and were unable to observe the complete development of *O. petrowi*, recovering only third-stage larva and adults. In order to better understand the development of *O. petrowi* larvae within both the intermediate and definitive host, this study expands upon research done by Kistler et al. [26] by providing a more effective means of infection and detailed examination of each stage of the *O. petrowi* life-cycle in both crickets and bobwhite.

## Methods

### Cricket colonies

Adult crickets were maintained in two standard 10-gallon glass aquariums (50.80 × 25.40 × 30.48 cm; hereafter, maintenance aquariums), which were fitted with screen aquarium covers (50.8 × 25.4 cm) to prevent escapes and high humidity, which is sub-optimal for adults [27]. A third 10-gallon aquarium was used as a rearing container for cricket nymphs (1st to 4th instars). Because cricket nymphs are vulnerable to desiccation [27], the rearing aquarium was covered with a tempered-glass lid (50.8 × 25.4 cm), which increased humidity and could withstand the temperatures generated by heat lamps used to warm the colonies. As an additional measure of safety, a welded wire screen was placed between the lid and heat lamp on the rearing aquarium to prevent the lamp from falling into the colony if the tempered-glass failed.

A heat lamp with an incandescent infrared bulb (120 V, 50 W) was set on top of each aquarium cover to attain temperatures (~ 30 °C) conducive for the development and upkeep of crickets [27]. Lamps were individually controlled by separate thermostats so that a daytime temperature of ~ 30 °C was maintained in each colony. In order to simulate a natural day-night cycle, a digital electric timer was set so that heat lamps were active from 8:00 h to 17:00 h, then switched off to allow colonies to cool to room temperature (~ 22 °C) at night. Photoperiod was maintained *via* automated lights on a 12:12 h light-dark cycle. Each aquarium was also fitted with a digital thermo-hygrometer which was monitored daily to ensure that daytime temperature in all aquariums was appropriate and humidity was 10–60% in maintenance aquariums and > 60% in the rearing aquarium. Colonies were lightly misted with a spray bottle if humidity fell below acceptable levels.

**Fig. 1:**
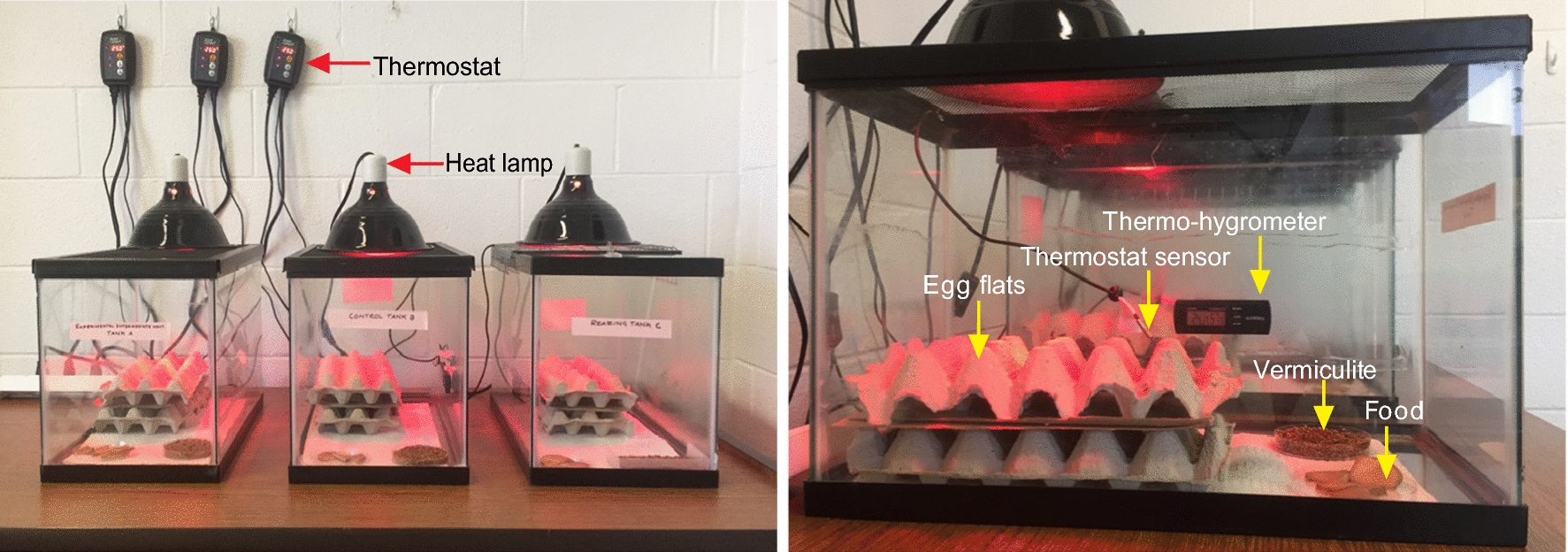
Complete setup for cricket (*Acheta domesticus*) colonies used in *Oxyspirura petrowi* life-cycle study

Several layers of cardboard egg flats were placed within each aquarium to provide shelter for crickets. Two layers of paper towels were used as substrate and these were replaced weekly to prevent fouling. Petri dishes filled with moist vermiculite were placed in maintenance aquariums to serve as egg pans in which female crickets could oviposit. A spray bottle was used to moisten the vermiculite as needed. Food was provided in plastic petri dishes that had a portion of the sidewall removed to allow crickets ease of access [27]. Crickets were fed *ad libitum* with Purina^©^ Game Bird Chow which was supplemented with cabbage leaves, orange and potato slices, all of which also provided water. Food was replaced every other day to prevent growth of bacteria and mold. The complete setup for cricket colonies is illustrated in Fig. 1.

### Experimental crickets

A total of 200 adult crickets were purchased from Fluker’s Cricket Farms, Louisiana. These crickets were divided equally between two maintenance aquariums and allowed to breed. Crickets were provided with egg pans, which were transferred into the rearing aquarium seven days after egg-laying was observed. Egg pans in the maintenance aquariums were immediately replaced so that crickets could continue to oviposit and these were transferred into the rearing aquarium and replaced at seven-day intervals until breeding ceased. Any remaining crickets from the initial colonies were discarded and maintenance aquariums were thoroughly cleaned with 10% bleach solution, rinsed with water and allowed to completely dry. Both maintenance aquariums were reset and prepared to house a cricket colony for experimental infection with *O. petrowi* (colony A) and an untreated control colony (colony B). Crickets hatched in the rearing aquarium and were allowed to develop up to the fourth-instar (~ 14 days), after which they were transferred into colonies A and B so that each colony had ~ 200 individuals. This ensured an adequate supply of crickets for the duration of the experiment, despite inevitable attrition within the colonies. Cricket colonies A and B were maintained as previously described for the duration of the experiment.

### Collection of parasite eggs

Eggs were collected from *O. petrowi* recovered from the eyes of wild bobwhite quail trapped at the Matador Wildlife Management Area in Cottle County, Texas, during September 2018. Bobwhite were trapped and necropsied as described by Dunham et al. [13] and gravid female *O. petrowi* were collected live in 1× PBS (Sigma-Aldrich, St. Louis, USA) solution. Worms were placed into a mortar and gently ground with a pestle to free the eggs from the reproductive tract [24]. Samples of 50 µl of 1× PBS containing *O. petrowi* eggs were examined under a light microscope (Olympus, Tokyo, Japan) at 40× magnification to ensure viability; of these ~ 96% were observed to be embryonated and only ~ 4% were non-embryonated and unviable.

### Infection of the intermediate host

In order to increase the likelihood that *O. petrowi* eggs would be ingested by crickets, intact and embryonated eggs from 10 worms were placed into a 1× PBS solution and applied to thin (~ 1 mm) slices of raw potato which had been lightly scored with a scalpel. The scoring allowed the solution containing eggs to better adhere to the surface of the slices and these were immediately fed to ~ 14 day-old crickets in colony A. Beginning on day post-exposure (DPE) five, crickets were dissected and examined for the presence of infective third-stage *O. petrowi* larvae according to Kistler et al. [26]. A total of three crickets from each colony were examined every two to three days until third-stage larvae were found. Afterwards, crickets were maintained for another seven days to ensure that *O. petrowi* developed to the third-stage before they were fed to bobwhite during the next phase of the experiment. All stages of parasite larvae recovered from crickets were examined at 40× magnification under a light microscope, with morphological characteristics described by Schwabe [24] being used to determine the developmental stages of *O. petrowi* within the intermediate host.

### Infection of the definitive host

The 30 pen-raised adult bobwhite used in this study were purchased from The Quail Ranch of Oklahoma (Wardville, Oklahoma) and maintained at the Wildlife Toxicology Laboratory (WTL) aviary. All bobwhite were individually housed, with 25 assigned to an experimental group and 5 to a control group. Prior to the experiment, both groups were allowed a 14-day acclimation period. During this time, fecal floats and PCR using eyeworm-specific primers were conducted to confirm no prior *O. petrowi* infection. When infecting the definitive host (DPE 0), each bird in the experimental group was fed four adult crickets from colony A, whereas control birds were each fed four adult crickets from colony B. Beginning on DPE seven, a bobwhite randomly selected from the experimental group was necropsied and examined for *O. petrowi* according to Dunham et al. [13]. If no *O. petrowi* were found, an additional bobwhite was necropsied. Necropsies were conducted at seven-day intervals until embryonated *O. petrowi* eggs were found in the feces of experimental bobwhite, after which all remaining birds from both groups were necropsied and examined for *O. petrowi*. To assess the development in the definitive host, all *O. petrowi* were collected in 1× PBS solution and examined microscopically at 40× magnification using morphological characteristics described by Schwabe [24] and Gibbons [28] to determine gender and developmental stage.

### Fecal floats

Fecal floats were done according to Kistler et al. [26] and were used to detect shedding of embryonated *O. petrowi* eggs, which would indicate the completion of the parasites life-cycle. Fecal samples were collected from all experimental birds every other day beginning on DPE 21 and continuing to DPE 31, after which they were collected daily until the end of the experiment. Prepared slides were examined for the presence of embryonated *O. petrowi* eggs under a light microscope at 40× magnification.

### PCR confirmation for different larval stages of *O. petrowi*

Genomic DNA was extracted from different larval stages of *O. petrowi* using Qiagen DNeasy Blood and Tissue Kit (Qiagen, Hilden, Germany) according to the manufacturer’s instructions. PCR was carried out using OXY_ITS2F (5′-CTT AGC GGT GGA TCA CTT GG-3′) and QEW_2578R (5′-AAC GTT ATT GTT GCC ATA TGC-3′) as described by Kistler et al. [26] and amplified products were visualized on 1.5% agarose gels using GelDoc XR+ (BioRad, Hercules, USA).

## Results

### Life-cycle of *O. petrowi* in crickets

**Fig. 2:**
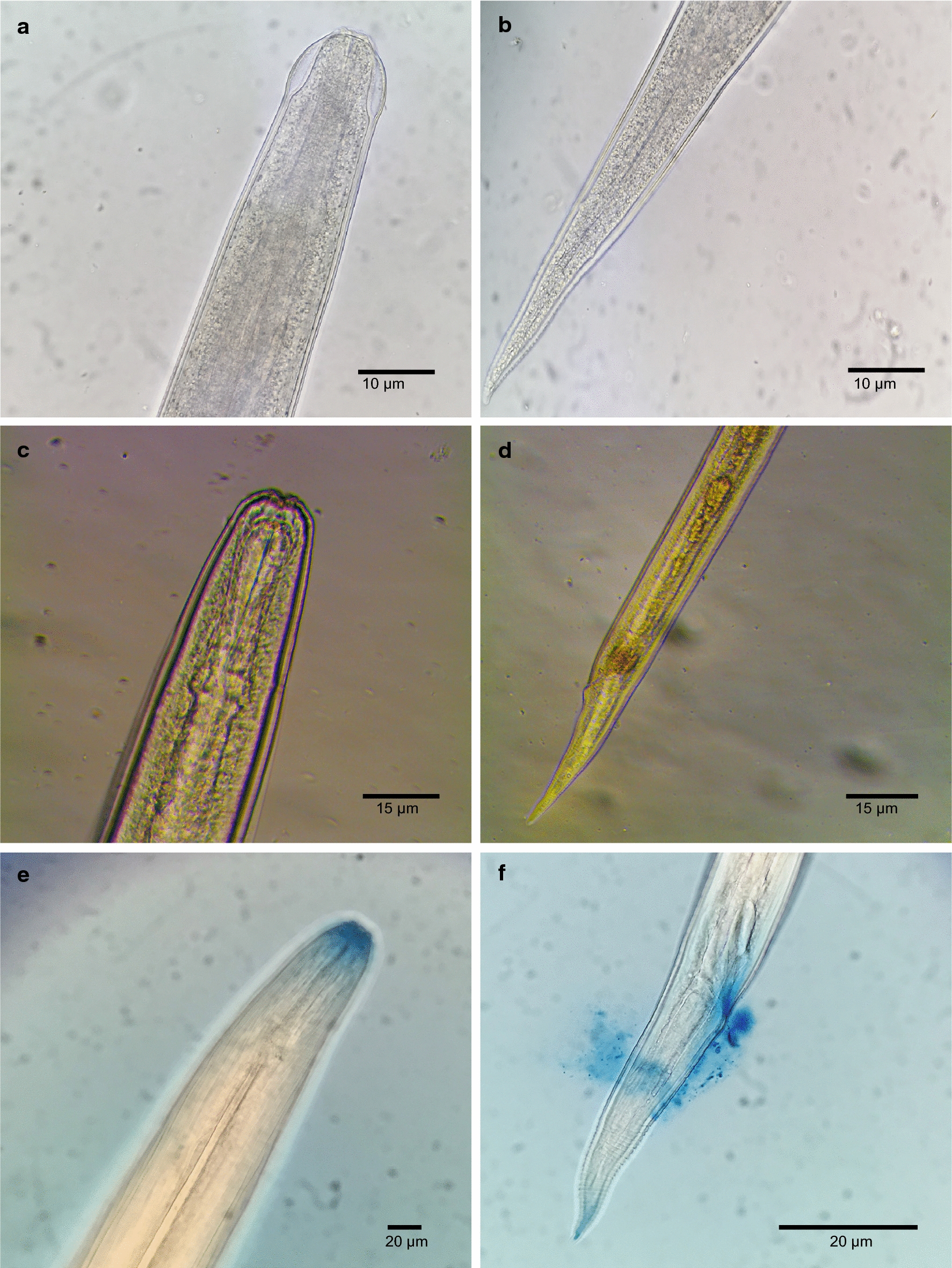
Different stages of *Oxyspirura petrowi* larvae recovered from experimental crickets (*Acheta domesticus*). **a** Anterior extremity of first-stage larva. **b** Posterior extremity of first-stage larva. **c** Anterior extremity of second-stage larva. **d** Posterior extremity of second-stage larva. **e** Anterior extremity of third-stage larva. **f** Posterior extremity of third-stage larva

**Fig. 3:**
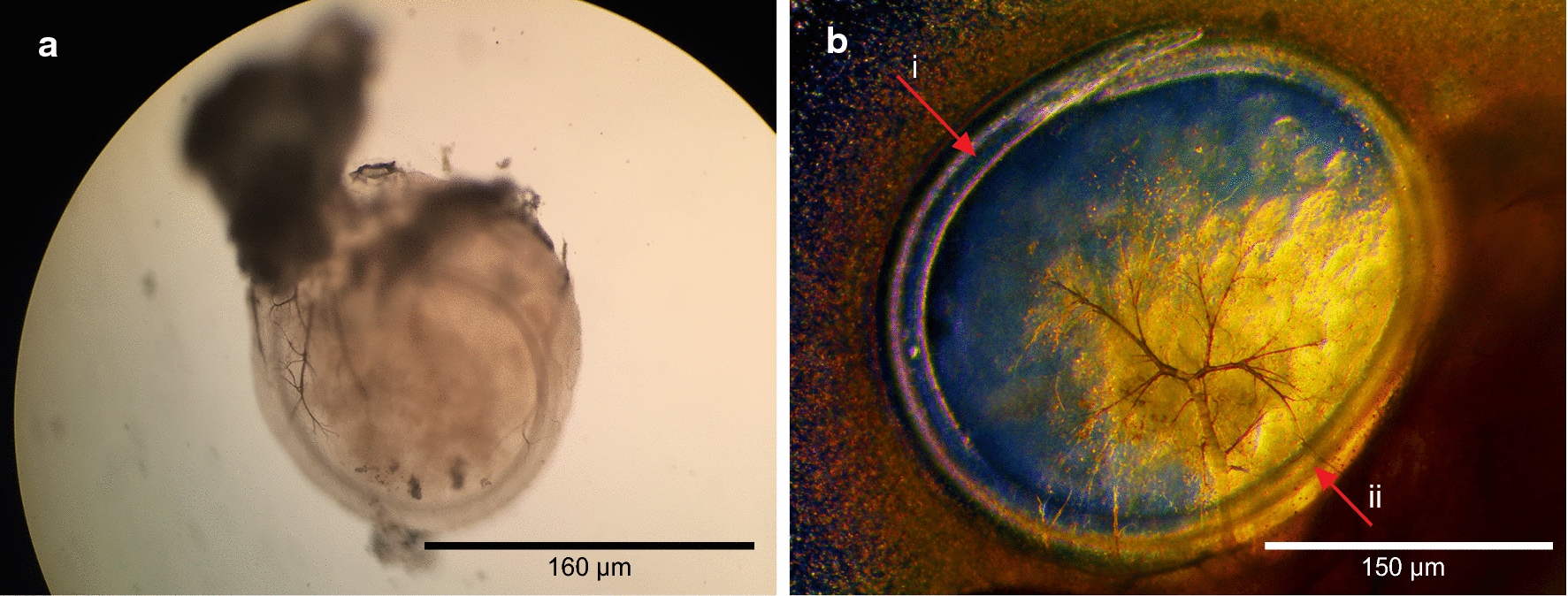
External view of encysted second-stage *Oxyspirura petrowi* larva. **a** Exterior view of the encysted larva. **b** Well-defined esophagus (i) and intestines (ii) of larva within the cyst

**Fig. 4:**
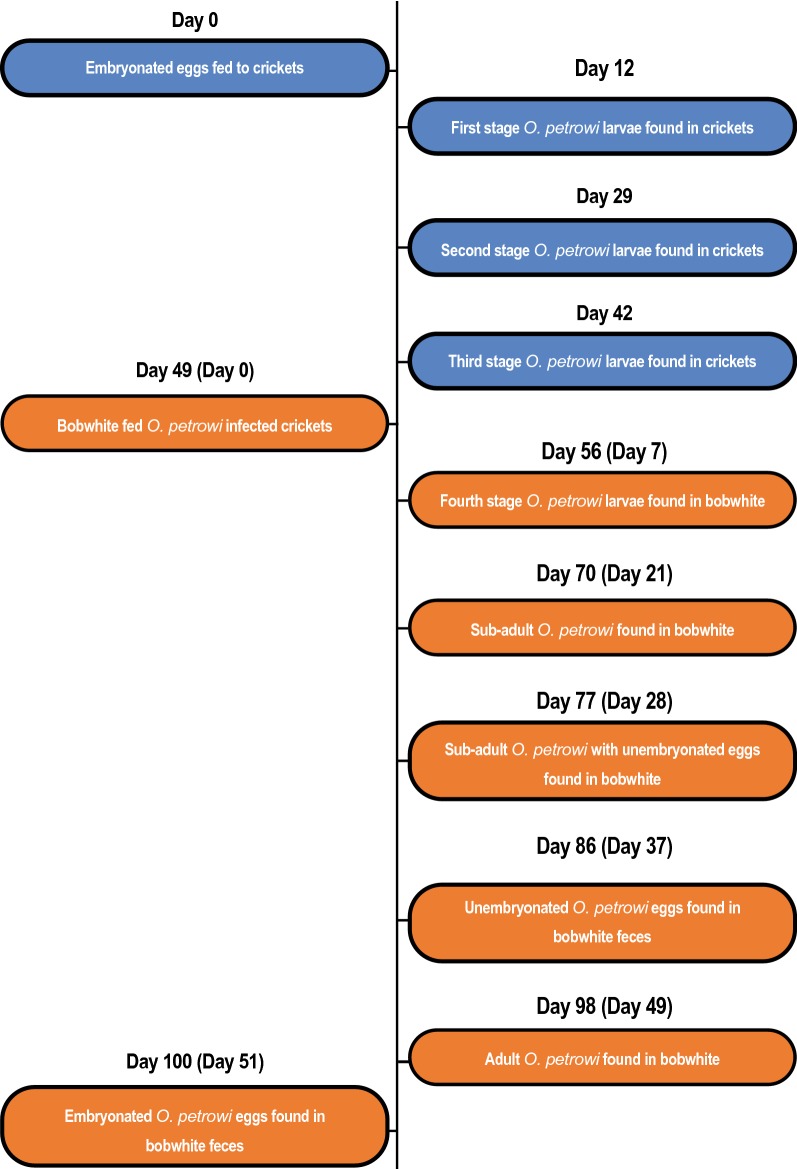
Timeline of complete *Oxyspirura petrowi* life-cycle. Duration spent within the intermediate host, the house cricket (*Acheta domesticus*) and the definitive host, the bobwhite (*Colinus virginianus*), are denoted by blue and orange, respectively

A total of 45 crickets from each colony were dissected and examined for *O. petrowi* larvae over the 42 days encompassing this phase of the study. No *O. petrowi* were found in any of the crickets examined from colony B. In contrast, 95.6% (43/45) of the crickets from colony A were found to be infected with a total of 113 *O. petrowi* larvae and infections ranged from 0–4 with an average of 2.51 ± 2.4 (SD) larvae per cricket. *Oxyspirura petrowi* larvae were first observed in crickets on DPE 12. Larvae were 145–235 µm in length and were determined to be first-stage based on their small size and indistinct internal organs (Fig. 2a). All of the first-stage larvae were located in the intestinal tracts of crickets. On DPE 29, the first encysted *O. petrowi* were found fused to the Malpighian tubules and intestines of crickets and these were identified as second-stage larvae by well-defined structures of the esophagus and intestine (Figs. 2b, 3). At this point, larvae had grown to 625–770 µm in length. *Oxyspirura petrowi* continued to be found within the Malpighian tubules and intestines of crickets and on DPE 42, third-stage larvae were first recorded in the intestinal region of crickets with specimens ranging between 7.0–8.5 mm in length. While morphologically similar to the second-stage larvae, the increased size and presence of cells surrounding the anterior end of the esophagus in the third-stage larvae (Fig. 2c) allowed for their positive identification. Additionally, six lobes were apparent on the anterior oral openings of third-stage *O. petrowi* larvae (Fig. 2c), a feature which was not visible in second-stage larvae. All stages of *O. petrowi* recovered from crickets were confirmed with PCR, showing amplification of *O. petrowi* ITS2 using specific primers [26]. Sequence analysis revealed that all larval stages of *O. petrowi* in the intermediate host had high identity (99.11–100%) to previous *O. petrowi* ITS2 sequences from the GenBank database KT958863-KF110800. The life-cycle of *O. petrowi* within the cricket intermediate host is illustrated in Fig. 4 and Table 1.

**Table 1:** Morphological differences of *O. petrowi* in intermediate and definitive host

Stage	DPI	Length	Morphological differences
Intermediate host			
First-stage (L1)	12	14–235 µm	Indistinct internal organs No clear nerve-rings Esophagus and intestine were not clear No excretory pore
Second-stage (L2)	29	625–770 µm	Well-developed esophagus and intestine
Third-stage (L3)	42	7.0–8.5 mm	Anterior of the esophagus surrounded by large number of cells Six lobes clearly seen on anterior oral opening
Definitive host			
Fourth-stage (L4)	7	10.6–11.7 mm	Absence of copulatory spicule in male and incomplete ovary in female Displacement in the intestine for development of reproductive organs in both male and female
Sub-adults	28	10.5–13.0 mm	Copulatory spicules in the male Female now carrying unembryonated eggs
Adults Male Female	49	13.5–14.3 mm 16.0–17.0 mm	Matured cuticular buccal capsule in both male and female Development of full reproductive organs: males exhibiting everted spicules and females carrying embryonated eggs within the oviduct Considerable difference between male and female length

*Abbreviations*: DPI, days post-infection

### Life-cycle of *O. petrowi* in bobwhite

**Fig. 5:**
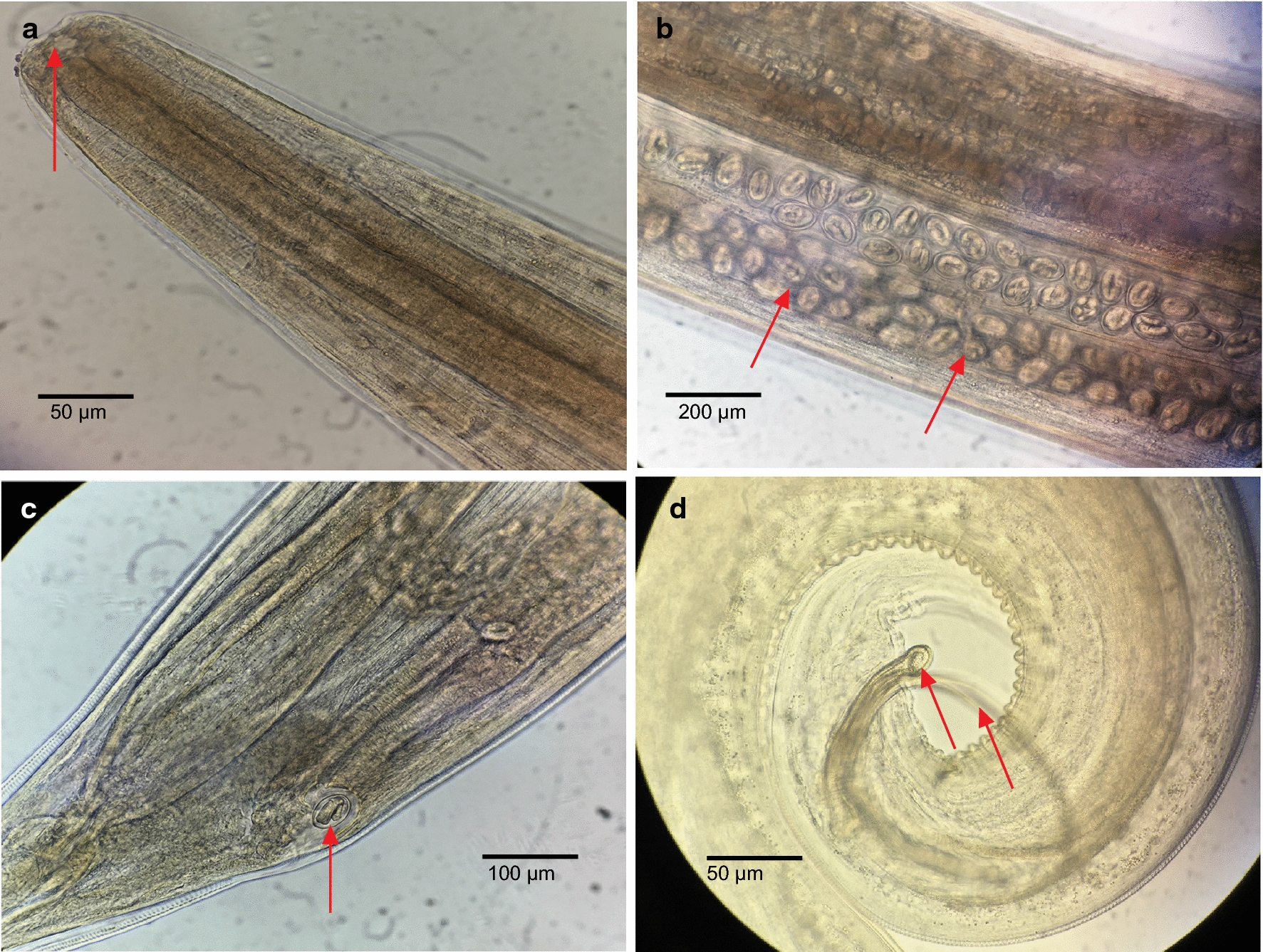
Morphology of adult male and female *Oxyspirura petrowi* recovered from experimental bobwhite (*Colinus virginianus*) infections. **a** Pronounced cuticular buccal capsule which is present in both males and females. **b** Embryonated eggs within the oviduct. **c** Vulva. **d** Two evaginated spicules

All birds were screened for *O. petrowi* infection prior to this study, with no eggs found in fecal floats and no PCR positives detected for *O. petrowi* ITS2 primers. During the 51-day period encompassing this phase of the study, 30 bobwhite were necropsied and examined for the presence of *O. petrowi*. While there was no evidence of infection in any of the five control birds, *O. petrowi* were found in 96% (24/25) of bobwhite from the experimental group. A total of 64 *O. petrowi* were recovered from these birds eye as reported earlier [9, 14] and infection ranged from 0–6 with an average of 2.56 ± 1.5 (SD) worms per bird. *Oxyspirura petrowi* were first recovered from the eyes of bobwhite on DPE 7; larvae were 10.6–11.7 mm in length and were identified as fourth-stage larvae based on the absence of the copulatory spicule in males and the incomplete development of ovaries in females (Fig. 2d). On DPE 14, fourth-stage larvae continued to be found in bobwhite, but the intestines in both male and female *O. petrowi* were noticeably displaced as the development of reproductive organs intensified. Then on DPE 21, the copulatory spicule was first observed in male *O. petrowi* and the overall length of worms had increased to 10.5–13 mm. *Oxyspirura petrowi* were considered to be sub-adults at this point, and unembryonated eggs were found within the oviducts of females by DPE 28. There were no significant morphological changes observed in *O. petrowi* over the next 14 days, although unembryonated eggs were found in bobwhite feces for the first time on DPE 37 and again on DPE 46. On DPE 49, the first adult *O. petrowi* were recovered from bobwhite. These specimens possessed fully developed reproductive organs with males exhibiting everted spicules and females carrying embryonated eggs within the oviducts. The cuticular buccal capsule of adult worms was more pronounced than that of sub-adults and the size of adults was also considerably larger, ranging between 13.5–14.3 mm and 16–17 mm in length for males and females, respectively (Fig. 5a, b). Finally, embryonated eggs were found within the feces of experimentally infected birds on DPE 51 indicating the completion of the *O. petrowi* life-cycle. Like the procedures with crickets, all stages of *O. petrowi* recovered from bobwhite and bobwhite feces were confirmed with PCR, showing amplification of *O. petrowi* ITS2 using specific primers [26]. BLAST analysis results revealed that sequences obtained from adult *O. petrowi* from crickets and bobwhites showed high identity (99.11–100%) to previous *O. petrowi* entries in GenBank database KT958863-KF110800. The life-cycle of *O. petrowi* within the bobwhite definitive host is illustrated in Fig. 4 and Table 1.

## Discussion

This study provides the first detailed representation of the complete development of *O. petrowi* in both the intermediate and definitive host. The use of pen-raised bobwhite with no prior exposure to *O. petrowi* made it unlikely that experimental birds were previously infected, which was substantiated by screening of all experimental birds and necropsies of untreated birds. Likewise, all crickets used in this study were bred to the second generation to ensure no carryover infection and dissections of untreated crickets confirmed that there was no coincidental infection with *O. petrowi*. Building on previous research by Kistler et al. [26], this study not only verified no prior *O. petrowi* infection in crickets and bobwhite, but also achieved nearly complete (96%) infection in both experimental crickets and bobwhite using a larger sample and greater resolution.

However, Kistler et al. [26] questioned the suitability of crickets as an intermediate host of *O. petrowi* due to low infections rates (16.6%) when crickets were fed gravid female worms, but others have questioned the suitability of *B. magna* as an intermediate host, as it is not a documented food source for bobwhite [7]. Additionally, a study using molecular techniques found a different cricket species, *Gryllus texensis*, to be a potential intermediate host but not *B. magna* [29]. Therefore, the house cricket is a suitable candidate to observe the infection dynamics of *O. petrowi* in a laboratory setting, as it is likely that these would be generally representative of those present in wild intermediate hosts while also providing a specimen that is easy to maintain in the laboratory. By changing the method of infecting crickets, a more successful and natural means of infecting crickets was achieved and resulted in a 95.6% infection rate. However, it is important to note that quantification of the initial dose of *O. petrowi* given to crickets was beyond the scope of this study. As such, future work is necessary to investigate the relationship between the quantity of *O. petrowi* eggs ingested and the magnitude of parasitism within the intermediate host.

Understanding the life-cycle of *O. petrowi* in both an intermediate host and the definitive host is essential to comprehension of how its life-cycle occurs in wild bobwhite. While there are multiple insect intermediate hosts of *O. petrowi* [25], this study only demonstrates the successful infection of crickets. However, the findings presented here can be used as a baseline to characterize the development of infection in other insect intermediate hosts. Although other variables, such as temperature, may influence the development of *O. petrowi* within the intermediate host, a greater degree of confidence is present regarding their development within bobwhite, as results from this study were similar to those discussed by Kistler et al. [26]. For example, embryonated eggs were found in infected bobwhite feces at DPE 51 in this study, which coincides with the DPE 52 reported by Kistler et al. [26]. Additionally, Kistler et al. [26] noted sexually mature *O. petrowi* at DPE 44–45 in bobwhite, whereas this study identified sexually mature *O. petrowi* at DPE 51. While these intervals are not markedly different, this does suggest that factors such as laboratory conditions and individual bobwhite response to infection play a role in the development of infection and should also be considered. Finally, while documenting the path by which *O. petrowi* moves into the eyes of bobwhite was beyond the scope of this study, we can infer that it utilizes a similar pathway to that of *O. mansoni* in poultry [24], as both nematodes are congeneric, utilize arthropod intermediate hosts, and infect galliform birds as definitive hosts. Although this research provides a detailed characterization of the entire life-cycle of *O. petrowi*, future work will be necessary to elucidate such physiological pathways of infection in both intermediate and definitive hosts.

## Conclusions

This study provides a detailed explanation of the sequential events in the *O. petrowi* life-cycle, focusing on the development of *O. petrowi* in both an insect intermediate host and avian definitive host. This research was able to demonstrate a 95.6% effective semi-natural means of infecting crickets with *O. petrowi* eggs, as well as a providing a comprehensive timeline of the subsequent development of larvae within crickets. This exemplifies the suitability of *A. domesticus* as an intermediate host for *O. petrowi*, providing researchers with a model for future evaluation of this parasites life-history using a widely available and easily maintained host, which is also likely to be representative of natural intermediate hosts of *O. petrowi*. Moreover, this is the first study to monitor and document the complete life-cycle of *O. petrowi* in the laboratory from egg to reproductive maturity, providing a clearer understanding of the timeframe associated with *O. petrowi* development in both of its hosts. The time points of infection reported herein may allow for better estimates into the potential for future epizootic events, as previously reported in the Rolling Plains Ecoregion [30]. Finally, this study adds to the increasing body of knowledge regarding the consequences of *O. petrowi* infection by providing a model for experimental infection to systematically examine the consequences of this parasite in bobwhite.

## Data Availability

The datasets used during the present study are available from the corresponding author upon reasonable request.

## Abbreviations

PBS: phosphate-buffered saline
SD: standard deviation
ITS: internal transcribed spacer
BLAST: Basic Local Alignment Search Tool
